# Induced Effects of Transcranial Magnetic Stimulation on the Autonomic Nervous System and the Cardiac Rhythm

**DOI:** 10.1155/2014/349718

**Published:** 2014-07-17

**Authors:** Mercedes Cabrerizo, Anastasio Cabrera, Juan O. Perez, Jesus de la Rua, Niovi Rojas, Qi Zhou, Alberto Pinzon-Ardila, Sergio M. Gonzalez-Arias, Malek Adjouadi

**Affiliations:** ^1^Center for Advanced Technology and Education, Department of Electrical and Computer Engineering, College of Engineering and Computing, Florida International University (FIU), USA; ^2^Neuroscience Consultants, USA; ^3^Baptist Health Neuroscience Center, Baptist Hospital of Miami, USA; ^4^FIU Herbert Wertheim College of Medicine, USA

## Abstract

Several standard protocols based on repetitive transcranial magnetic stimulation (rTMS) have been employed for treatment of a variety of neurological disorders. Despite their advantages in patients that are retractable to medication, there is a lack of knowledge about the effects of rTMS on the autonomic nervous system that controls the cardiovascular system. Current understanding suggests that the shape of the so-called QRS complex together with the size of the different segments and intervals between the PQRST deflections of the heart could predict the nature of the different arrhythmias and ailments affecting the heart. This preliminary study involving 10 normal subjects from 20 to 30 years of age demonstrated that rTMS can induce changes in the heart rhythm. The autonomic activity that controls the cardiac rhythm was indeed altered by an rTMS session targeting the motor cortex using intensity below the subject's motor threshold and lasting no more than 5 minutes. The rTMS activation resulted in a reduction of the *RR* intervals (cardioacceleration) in most cases. Most of these cases also showed significant changes in the Poincare plot descriptor SD2 (long-term variability), the area under the low frequency (LF) power spectrum density curve, and the low frequency to high frequency (LF/HF) ratio. The *RR* intervals changed significantly in specific instants of time during rTMS activation showing either heart rate acceleration or heart rate deceleration.

## 1. Introduction

**Figure 1:**
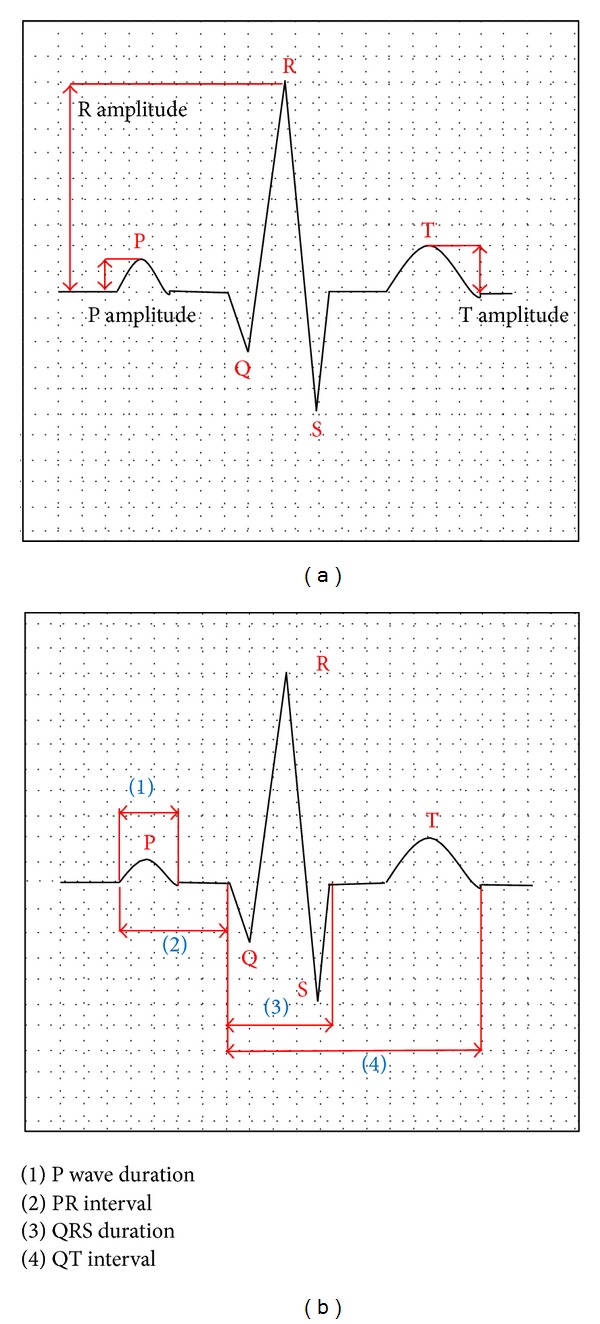
PQRST waveforms from an electrocardiography device.

The TMS technology was introduced in the 1980s, and since its introduction, it has been used in clinical care for several neurological disorders [1–4]. The initial intent of this technology was to improve the health of patients with depression as exemplified in studies [5–8]. Its application has now been extended to gauge the merits of magnetic stimulation to other neurological disorders such as epilepsy [9–11], Huntington's disease [12], Parkinson's disease [13], different effects of schizophrenia [14–16], Alzheimer's disease, and effects of aging [17, 18], in patients who have had a stroke [19–21], autism [22], and attention deficit and hyperactivity disorders [23, 24]. These are by no means an exhaustive listing of such noteworthy references, but these are examples of studies that highlight the extensive use of TMS technology. It should be noted that the use of TMS can be performed under two modes of operation, namely, single pulse [25] or repetitive mode of stimulation [26, 27]. Safety measures and ethical considerations in the use of TMS technology are well described in [28, 29]. In many of these disorders, the autonomic symptoms are peculiar and may represent the clinical onset of the disorder. For instance, motor activity and some brain abnormalities are associated with changes in the heart rate rhythm and blood pressure; among those abnormal conditions are epilepsy, stroke, and intense emotional stress. External stimulations with TMS are accompanied with diverse effects depending on the site of stimulation [28, 29]. When applied to the motor cortex, which is accompanied by the contraction of muscles, TMS can alter the heart rate variability (HRV) due to connections between the brain cortex and the autonomic centers [30, 31]. Generally, central nervous system (CNS) activation of motor areas is accompanied by diverse scales of cardiac acceleration mediated by the autonomic nervous system (ANS). The ANS is in turn modified by the reflex activity triggered by feedback of the cardiovascular system and articulation sensors which are stimulated by movement [32]. The brain cortex, the brain stem, and the autonomic nerves can alter the heart function and potentially trigger arrhythmias [33]. Such clinical manifestations in some patients suggest that there is a link between cortical structures and autonomic centers. However, not too many studies refer to this problem; only very few research groups have investigated this phenomenon [29]. For example, it is known that epilepsy alters significantly the heart rhythm [10] and produces prolonged QT intervals, T wave alternans, and ventricular late potentials. During seizures, bradycardia and asystole states can occur in some patients. Stroke can also alter the heart rhythm [34] and intense emotions can disrupt significantly the heart rate and blood pressure of a given patient [35]. An illustration of the PQRST deflections of the heart is given in Figure 1.

High frequency stimulation with rTMS (≥5 Hz) produces cortical excitation, so when applied to the primary motor cortex, it additionally provokes muscle movement. It could also evoke cardiac responses mediated by connections in the brain cortex with the cardiac-related centers of the CNS. In several studies, muscle reaction due to rTMS over the left primary motor cortex (M1) appears to be limited to the limb areas; however they are also accompanied by changes in the heart rate variability (HRV) of the *RR* intervals and the power spectrum of the ECG signals. By using low frequency rTMS [36], it was found that the low frequency (LF) and high frequency (HF) power were significantly increased. Also by measuring the HRV, it was found that rTMS produced significantly greater reduction in the sympathetic/parasympathetic ratio, suggesting improvement in the sympathovagal balance. The LF and HF areas from power spectral plots show that there is an increment of these values after rTMS stimulation, while the LF/HF ratio decreased [37]. As rTMS technology develops in scope and application domains, its use on patients with known cardiac conditions should be carefully weighed with respect to the heart ailment itself and the effects that were observed in this study with healthy controls. With this posed assertion, this study presents a new methodology that relies on a newly developed hardware-software assimilated system with real time integration of electrocardiography (ECG) recordings while a patient is undergoing brain stimulation through rTMS. Real time effects of rTMS on the HRV are performed both in the time and frequency domains through the automatic examination of the *RR* intervals variability [17].

The integration of several modalities augments the capabilities of a given system to produce a more accurate diagnosis and therefore a better plan for treatment [16, 38–40]. The proposed study thus aligns in time and space electrocardiography (ECG) with the neuronavigated transcranial magnetic stimulation (TMS) machine using a repetitive pulse (rTMS) [41]. By time and space alignment we mean the opportunity for simultaneous recordings of the ECG under repeated transcranial magnetic stimulation (rTMS) while using the same 3D coordinate system on the same patient.

## 2. Materials and Methods

### 2.1. Specific Aim of the Study

The strategy behind this study design is to control the magnetic stimulation of the brain according to the selected moments of the cardiac cycle. Empirical evidence suggests that if the magnetic stimulus is not adequately synchronized with the cardiac cycle, there is potential for slowing the heart rate; however, the same stimulus can produce minimal or no alteration of the heart rate if an adequate synchronization is carefully chosen.

More importantly, experimental evaluations indicate the importance of the interval between two consecutive *R* deflections (the deflection with the highest amplitude in the cardiac cycle), which is referred to in this study as the *RR* interval. Whether the treatment is through providing stimulation or medication, every precaution needs to be taken such that any effects observed on these heart deflections in healthy control subjects could not otherwise yield unwarranted effects on subjects with specific heart ailments. For example, according to the American Heart Association, ADD/ADHD stimulant medications have been found to cause sudden death in children and adults with specific heart conditions. Several research studies have later shown that these medications do increase the heart rate in some predisposed individuals. Even though such side effects are rare and are observed in a small number of children with ADHD, they remain of extreme importance and every precaution should be taken to prevent these types of risks.

### 2.2. Subjects

The effects of the high frequency repetitive TMS (rTMS) on 10 young volunteers (3 females and 7 males) with no history of medical conditions were examined. The study was approved by the Institutional Review Board (Protocol number: IRB-13-0230; Reference number: 101219) and consent forms were provided to the subjects. These subjects did not experience any signs of any cardiovascular disease and were not taking any medication and were advised not to take any caffeine or perform any physical activity that can alter the ECG signal prior to the rTMS session.

All the subjects were laid down in a comfortable chair in a supine position. The Nexstim system's 6-channel EMG module (SR = 1450 Hz, cut-off frequency of 350 Hz for the low pass filter) automatically calculated the motor evoked potential (MEP) amplitudes and latencies as the motor cortex (cortex area of the thumb) is stimulated [34]. Disposable Ag-AgCL surface electrodes were used to record the MEP responses that were displayed in a computer screen in order to assess the validity of the response based on the strength of MEPs reflecting the ability of that area to develop muscle contraction.

### 2.3. Design and Implementation of the Study

The research aims of this study were carried out using a hardware-software system developed in our lab that aligns in time and space ECG with the operational functions of the TMS machine. This integrated and noninvasive ECG-TMS system consists of two main components: (1) a novel hardware design solution that automatically activates the solenoids of the TMS pedals for the 3 different operational functions: increasing the intensity, decreasing the intensity, and triggering the electromagnetic pulse; (2) a software module that serves a dual purpose: (i) reading the ECG signal and synchronizing the trigger of the TMS via the hardware component, a synchronization which can be made in relation to any of the deflections of the recorded ECG in monitoring the heartbeat during brain stimulation [42], and (ii) serving as a graphical user interface for man-machine interaction and for the potential deployment of a feedback mechanism.

The magnetic stimulation was performed using the Nexstim eXimia TMS system. The rTMS session was delivered by an 8-inch coil with an orientation quasi-perpendicular to the area of the central sulcus (about 45° from the brain midline) and applied to the left primary motor cortex (M1), which is related to the hand movement of the right side. The coil was positioned 2 cm lateral to the scalp projection of the sagittal suture and 1 cm to the projection of the coronal suture (posterior portion of the frontal lobe). The motor threshold was calculated for each subject prior to the session. This threshold was defined as the lowest magnetic stimulation possible that was still able to induce MEPs response in the range of 100–500 *μ*V peak-to-peak amplitude in the right arm in the abductor pollicis brevis (APB) muscle in at least 4 out of several attempts. The MRI-guided marker was positioned on the motor cortex at approximately 25 mm depth as an initial position to begin the stimulation. Then, magnetic pulses with an intensity of 10% below this motor threshold were applied and ECG and blood pressure were recorded before, during, and after the rTMS sessions. Heart rate variability (HRV) was processed in the time domain. A comparison was established between the baseline and rTMS activation recordings.

The ECG biophysical amplifier used was able to record 12 leads of real time ECG at a sampling rate (SR) of 1 KHz. A band-pass filter (0.05–300 Hz) was implemented and applied to the recorded signals with a 16-bit A/D conversion, a sensitivity of 0.4 *μ*V, and a common-mode rejection ratio (CMRR) of 120 dB.

**Figure 2:**
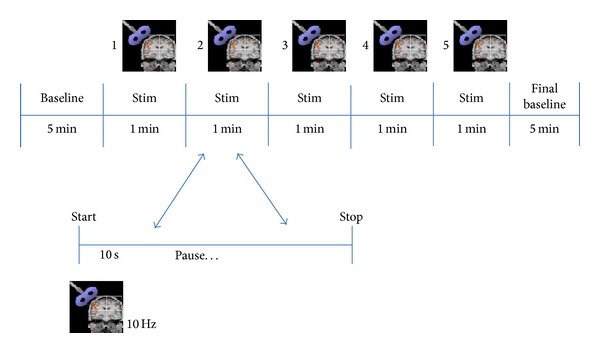
rTMS protocol implemented for all subjects. Note that 5 stimuli were applied during intervals of 1 minute of duration.

The rTMS protocol implemented for all subjects is as described in Figure 2, where trains of stimuli of 1-second duration at 10 Hz were applied to four different electrode locations (*F*1*L*, *F*2*L*, *F*1*R*, and *F*2*R*) on the scalp; as a total, 50 pulses were delivered to each brain location, as the train of pulses was repeated 5 times at intervals of 1 minute (interstimulus time: 59 seconds). The intensity of the magnetic pulses was below the visual motor response in the hand. An initial blood pressure was obtained and an ECG signal was recorded for 5 minutes as baseline measurements. These same measurements were also collected at the end of a 5-minute session of brain stimulation to assess the difference in these measurements between baseline and after brain stimulation.

**Figure 3:**
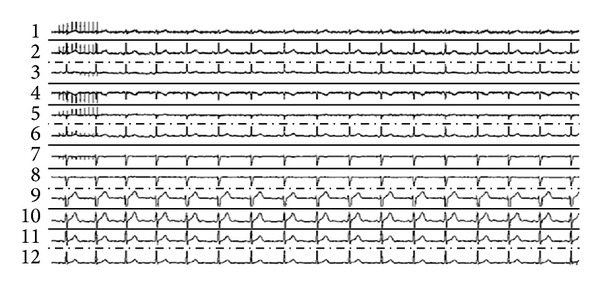
12-lead ECG recording. Note: stimuli artifact (1 sec, 10 Hz) is seen at the beginning of recording in several leads.

The TMS session applied to the subject in order to record the ECG signals was delivered by the in-house developed software, which is fully compatible with and coupled effectively to the TMS machine. An extended ECG montage with 12 leads was simultaneously recorded as illustrated in Figure 3 and stored in a computer, making it amenable to real time ECG monitoring. During the offline analysis of the ECG, normal *RR* intervals were automatically identified, removing those intervals altered by noise or by a surge of ectopic beats, a disturbance of the cardiac rhythm. These intervals were replaced by a mean *RR* interval value calculated automatically by the software.

**Figure 4:**
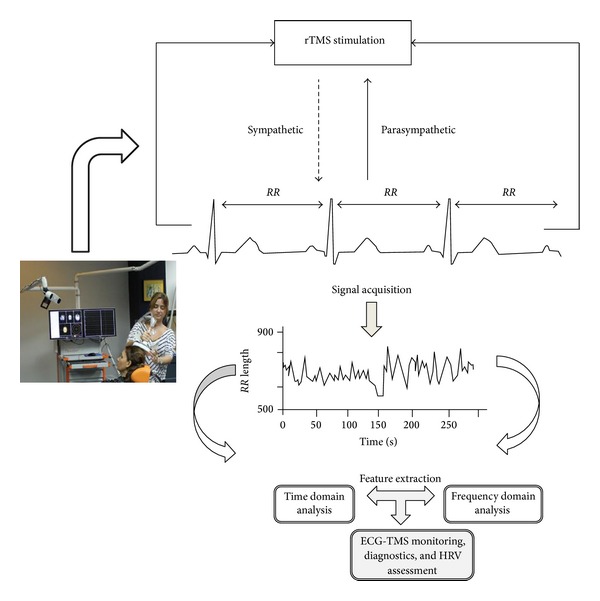
Design structure of the ECG-TMS system.

Figure 4 describes the main steps of the proposed study. A high frequency magnetic pulse was applied to the subject in the motor cortex region in order to see the induced changes of this stimulation in the cardiovascular system and to gauge the balance between the sympathetic and parasympathetic nervous system. The ECG signal was acquired and digitized for 15 minutes. Feature extraction algorithms were performed on the input signal, and a reliable *RR* interval vector was extracted for further processing in both time and frequency domains.

### 2.4. Time Domain Variability

Time domain variables such as mean, standard deviation of *RR* intervals, and coefficient of variation measured before, during, and after stimulation were extracted [43]. A Poincare plot representation and calculation of its parameters such as SD1 (short-term variability) and SD2 (long-term variability) were also assessed [44].

**Figure 5:**
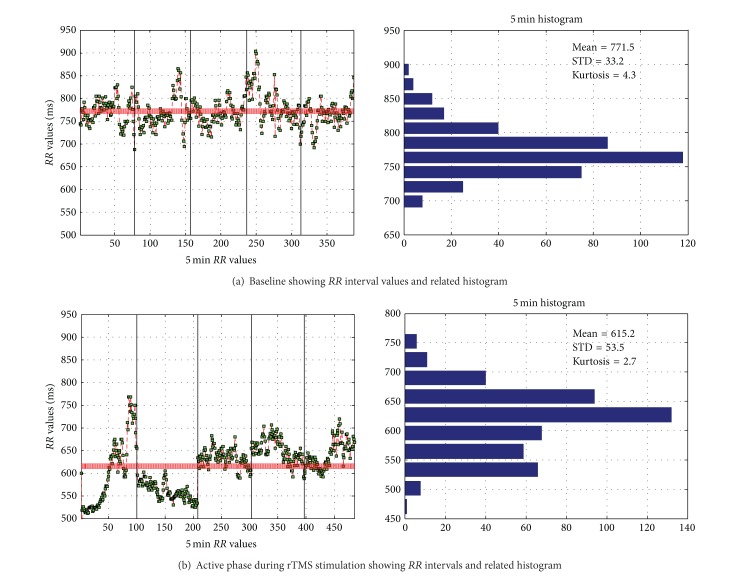
*RR* interval and related histograms comparing (a) baseline to (b) active phase.

The heart rate variability (HRV) was significantly changed, as shown in Figure 5 for a particular subject. In this figure, vertical black lines divide the session into five 1-minute intervals. An increase of the heart rate was observed as the *RR* interval values decreased due to the stimulation on the left frontal motor cortex. Specifically to this subject, during the first minute, there was an evident decrease of the *RR* interval values in the active phase when the subject is stimulated using rTMS as compared to the baseline when the subject is at rest.

As can be observed from the histograms of Figure 5, the mean of the *RR* interval value dropped from 771 in the baseline phase to 615 during the stimulation phase. Furthermore, the standard deviation increased from 33.2 at baseline to 53.5 during stimulation.

**Figure 6:**
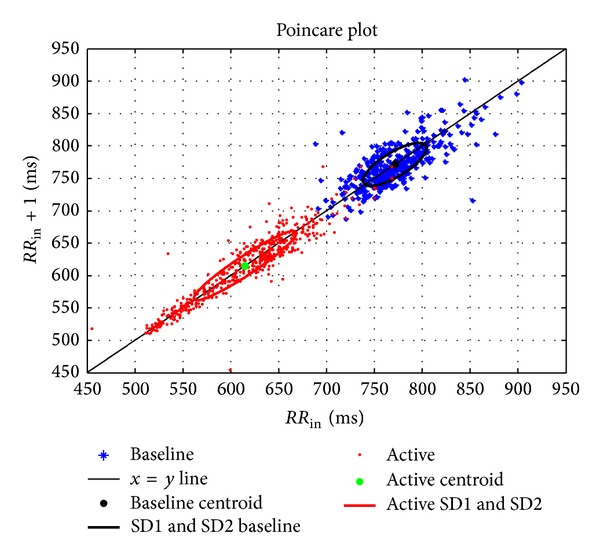
Poincare plot of *RR* intervals distribution.

To elicit a better understanding of these *RR* intervals, Figure 6 represents a Poincare plot with the distribution of the *RR* interval values at baseline and during stimulation performed on the left side of the motor cortex. The line (*x* = *y*) in the plot has a physiological significance because all the points that fall in this line correspond to equal and consecutive *RR* interval values (distances from *R*_(1)_-*R*_(2)_, *R*_(2)_-*R*_(3)_, and so on until *R*_(*n*−1)_-*R*_(*n*)_). All the points above the identity line correspond to a decrease in the heart rate and the points below this line correspond to an increase in the heart rate [44].

The Poincare plot for a given *RR* vector of length *N*, denoted by *X* = (*x*_1_, *x*_2_,…, *x*_*N*_), can be derived using the following two subvectors:

(1) $$\begin{array}{c} X_{RR}=\left(x_{1},x_{2},\ldots ,x_{N-1\;}\right),\;\;X_{RR+1\;}=\left(x_{2},x_{3},\ldots ,x_{N\;}\right). \end{array}$$

These two subvectors correspond to the *x*-axis and *y*-axis of the Poincare plot. When this graphical representation is used with real data (10 minutes of ECG recording), the data points are fit to an ellipse for further interpretation. This plot is characterized by two standard descriptors (SD1, SD2) as defined below:

(2) $$\begin{array}{c} \text{SD}1=\sqrt{\text{var}\left(X_{RR}\right)},\;\;\text{SD}2=\sqrt{\text{var}\left(X_{RR+1\;}\right)}. \end{array}$$

The HRV measure can be expressed as in

(3) $$\begin{array}{c} \text{var}={\left(\frac{X_{RR\;}}{X_{RR+1}}-\mu \left(\frac{X_{RR}}{X_{RR+1}}\right)\right)}^{2}. \end{array}$$

An ellipse has two perpendicular axes that intersect at the center of the ellipse due to its symmetry. The larger of these two axes is called the major axis (SD2), while the smaller of these two axes is called the minor axis (SD1). It is considered that SD1 reflects the standard deviation of the short-term variability of the ECG, while SD2 reflects the standard deviation of the long-term variability of the ECG.

A clear displacement and higher concentration of the points (shorter *RR* interval) are observed during stimulation as recorded from the *F*1*L* electrode, which is located on the left hemisphere. The SD2 descriptor increased and SD1 decreased, which is clear evidence of the change in both the short- and long-term variability.

The Poincare plot and its inherent descriptors constitute a novel approach to visualize the HRV in a given patient. The mean and standard deviation of these descriptors were also calculated for all subjects at baseline and during stimulation in order to assess a meaningful global change within the two different phases. Quantified results are provided in Table 1.

An overall assessment of these results as given in Table 1 for all the subjects indicates that the average of the *RR* intervals during the entire rTMS session decreased (baseline 862 ± 94, rTMS 840 ± 16 ms). These results also show a slight increase of the heart rate (70 to 71 bpm). However in 7 out of the 10 cases, *RR* intervals decreased (cardioacceleration; from baseline 850 ± 86 to rTMS 830 ± 119 ms), while, for the remaining 3 cases, *RR* intervals increased (cardioinhibition; baseline 826 ± 137, rTMS 865 ± 108 ms).

A new descriptor was incorporated in order to assess with higher accuracy the total variability of a session. The new descriptor takes into account the two standard deviations of the two axes (SD1 and SD2) and the average behavior of the heart rate (Mean_*RR*_) as a direct measurement of the HRV. This measurement is normalized with respect to the number of subjects (*N*) as follows:

(4) $$\begin{array}{c} {\text{Total}}_{\text{var}}=\frac{{\text{Mean}}_{RR}*\text{SD}1*\text{SD}2}{N}. \end{array}$$

It is observed that the SD1 descriptor did not change after rTMS (baseline 32 ± 9.5 and rTMS 30 ± 11 ms). The descriptor SD2, on the other hand, had a more pronounced variation (baseline 71 ± 17, rTMS 81 ± 17 ms). SD1 in 70% of the cases showed the same trend (baseline 30 ± 10, rTMS 30 ± 12 ms). SD2 showed again a larger increase (baseline 64 ± 15, rTMS 80 ± 17 ms). Conversely in 30% of the cases, both descriptors, SD1 and SD2, decreased after rTMS (SD1 baseline 36 ± 11, rTMS 31 ± 4.3; SD2 baseline 88 ± 11, rTMS 82 ± 19 ms).

The periodogram slope of every minute interval of the 5-minute period of stimulation was also calculated. If the trend of the periodogram plot increased, the slope of the best fit line was quantified as “+1”; if instead the trend of the periodogram plot decreased (decrease in *RR* intervals), the slope was quantified as “−1”; if no change (no significant increase or decrease of *RR* intervals) occurred, it was quantified as “0.” 

**Figure 7:**
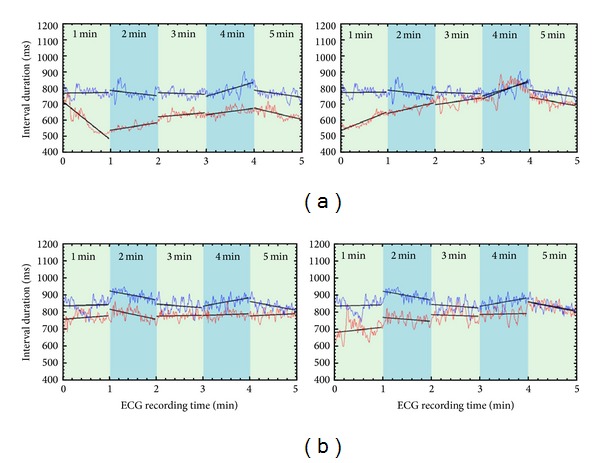
Illustrative examples on observed changes on the *RR* intervals for 2 subjects: subject 1 (a); subject 2 (b).

Figure 7 shows a comparison between baseline (supine position during 5 minutes) and the activation phase (during rTMS stimulation using a frequency of 10 Hz). The slopes of the baseline phase (blue) and active phase (red) of the *RR* intervals are calculated for each minute during the recording to show the effect of the rTMS on the heart rate.

The stimulation started at the beginning of every minute and lasted only 1 second. The effects of the magnetic stimulation are observed for 59 seconds, until another stimulation of 1 second begins. From these results, it can be observed that there is an evident deflection of the *RR* intervals from the baseline, and during stimulation, the heart rate increased due to a decrease of the *RR* interval values.

An interesting remark to be made on the basis of the results shown in Figure 7 is the gradual move of the red segments towards the blue (baseline) segments in time as stimulations are given. Does this mean that in time the effect of the stimulation on the ECG is lessened? In other words it is as if one is startled by such stimulation at first and then gets used to it in time; it is an observation to be considered in future studies.

The results showed that changing of trends in slopes during rTMS was statistically significant regardless of whether the orientation is positive or negative. Figure 7 also shows that the number of slope changes, positive or negative, was more frequent at any minute of rTMS than at baseline. These findings suggest that there was disruption of the vagosympathetic balance in most cases as a consequence of rTMS. Some cases showed cardioacceleration followed by cardioinhibition as shown in Table 2.

### 2.5. Statistical Analysis

Comparisons of average values of *RR* intervals in the whole 5-minute recording were made using the Student's* t*-test for small samples. When recording signals are analyzed empirically, it is mandatory to assess if results are consistent or are only due to random events. This is performed by statistical hypothesis testing using the *P* value, which is the probability of obtaining the observed test statistics given the null hypothesis. If the *P* value is smaller (a given predefined significance level), the null hypothesis is rejected and the observed result is considered “significant” for our analysis. To compare slopes changes, three categories were considered: no change (0), positive (+1), or negative (−1). Statistical significance of *P* < 0.05 using the chi-square method was considered. The chi-square statistic measures, instead of a population average, the difference between the observed counts and the counts that would be expected if there was no relationship between the two groups (baseline and stimulation). An important observation that can be made from the results shown in Table 2 is that, for the baseline, the 3rd minute (indicated by a ∗) is the one that showed the most significant variation in terms of changes in slope trend (*P* < 0.05). In the active phase, it is minute 5 (indicated by a ∗∗) that showed the most significant variation in terms of changes in slope trend (*P* < 0.02).

### 2.6. Frequency Domain Variability

Spectral analysis has been performed using the fast Fourier transform (FFT) in the tachogram signal. For frequency domain measurements, it is recommended that the duration of the ECG recording is at least greater than 5 minutes. ECG signals were visually corrected for ectopic and missed beats. This was performed by filtering the signal to eliminate the false peaks and by interpolating in between missing beats. This way a modified and corrected tachogram is obtained for the analysis. The power spectrum of the HRV vector during 5 minutes of ECG recording was used as a quantitative measurement to assess autonomic changes in the cardiovascular system.

In humans there are two frequency ranges of interest defined in the low frequency as LF = (0.04–0.15 Hz) and in the high frequency as HF = (0.15–0.4 Hz). Parasympathetic and sympathetic effects are associated with the changes of these frequencies. Parasympathetic activity is considered responsible for these HF values. Both parasympathetic and sympathetic activities, together with other mechanisms, are considered to determine the LF range [45, 46].

**Figure 8:**
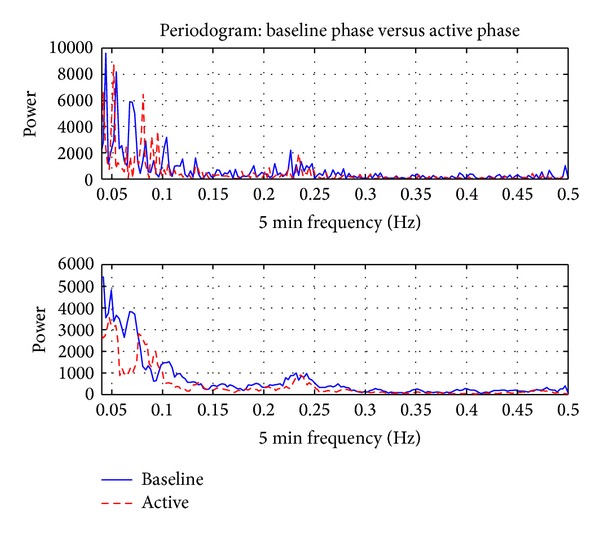
Power spectrum. Note: sampling rate of the ECG was 1 Hz, so the frequency spectrum was plotted until 0.5 HZ (Nyquist frequency criteria). There is an increment of the power around 0.05 and 0.1 Hz during the stimulation using 10 Hz and 5 repetitions.

As shown in Figure 8, the results of a representative subject show some differences in HRV as determined from spectral analysis in the LF and HF ranges. These results are observed for all subjects of the study. Repetitive TMS, particularly after stimulation of the left hemisphere, induced a slight decrease in the parasympathetic (HF components of the spectrum) and a stronger decrease in the LF power spectrum (partially sympathetic activity). The quantitative changes in the power spectrum of the HRV proved that the cardiovascular control mechanism was altered during rTMS [47, 48].

**Figure 9:**
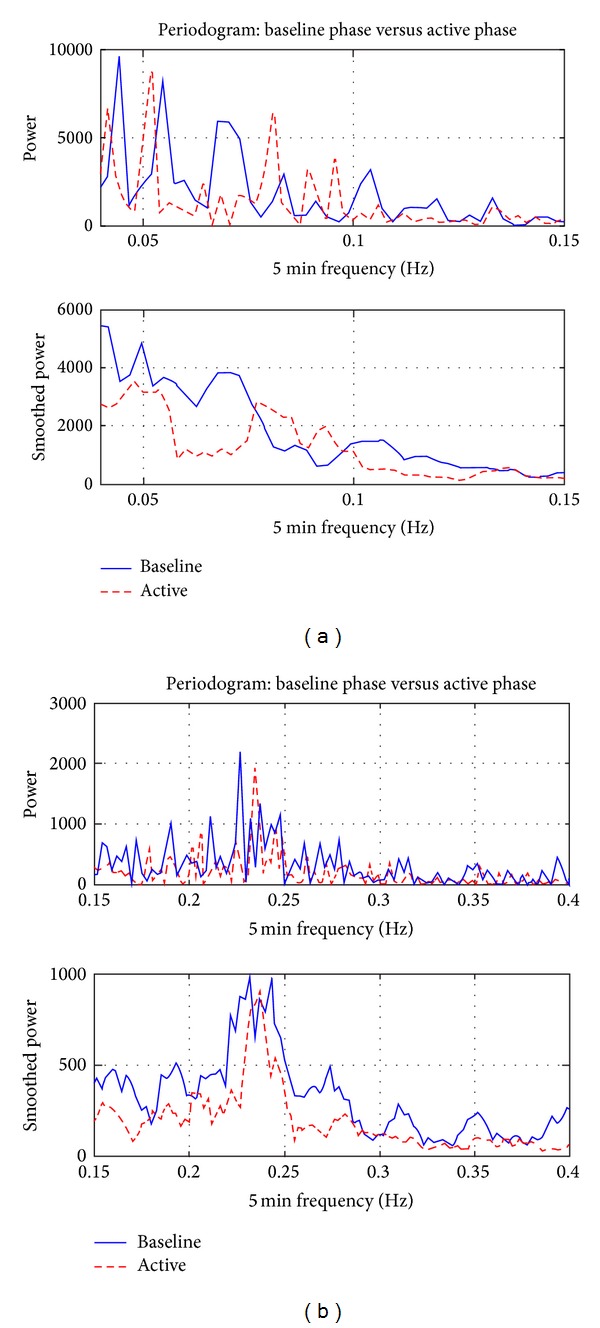
Power spectrum: (a) corresponds to LF and (b) to HF components.

In reference to the results shown in Figure 9 and Table 3, frequency domain estimates (see Table 3) suggest that the ratio LF/HF, an indicator of sympathetic activation, increased for 60% of the subjects while it decreased for the remaining 40% of the subjects. The majority of these results indicate that rTMS influenced the vasomotor center, which is located in the reticular substance of the medulla and pons, connected with the motor cortex. The indirect influence of rTMS upon cardiac centers was apparently heterogeneous. Some previous reports describe changes in the power spectrum of the HRV accompanying rTMS. For example, when using low frequency rTMS it was found that both LF and HF power increased significantly. The area under the LF and HF curves from power spectral plots increased after TMS stimulation and LF/HF ratio decrease for 40% of the subjects.

**Figure 10:**
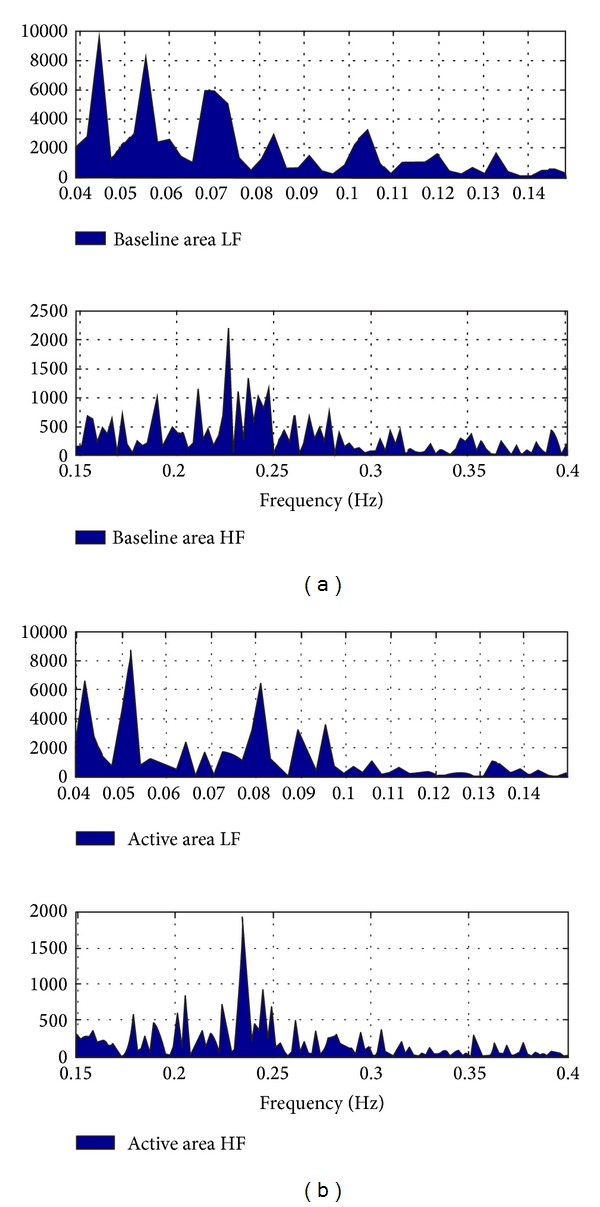
Area under the curve: baseline (a) and active phase (b).

A comparison of the area under the curve (AUC) of the LF and HF components, as observed in Figure 9, was performed on the spectral curve of the *RR* intervals during 5 minutes (at baseline and in the activation phase) for one of the subjects as an illustrative example. Results shown in Figure 10 indicate that there is a considerable change in the total area when comparing baseline to the activation phase: total baseline HF = 81 and total activation HF = 48. The LF is also altered during stimulation.

## 3. Discussions

No adverse incidents and no relevant changes in blood pressure (±10 mmHg in systolic or diastolic pressure), or any discomfort, were expressed by the subjects under rTMS stimulation. Motor responses observed in some cases were hand contractions of the contralateral side stimulated. Results show that, after rTMS, the mean *RR* interval decreased by 2% (cardioacceleration). Two distinct groups were identified according to their reaction to rTMS: group 1 with cardioacceleration (7 cases) and group 2 with cardioinhibition (3 cases). In group 1, *RR* intervals decreased by 6%; one case showed a heart rate increment of 10 bpm. In this group, the SD2 descriptor of the Poincare plot increased by 5.4% while SD1 did not change. The area under the curve of the low frequency band (LF) of the power spectrum density increased by 4%, while the high frequency band increased by 8%. The ratio LF/HF increased from 0.94 to 1.02. In group 2, an increment of 5.2% in *RR* interval (cardioinhibition) was observed. In this group, both SD2 and SD1 decreased. The LF band area was decreased by 17%, while the HF band increased by 14%. However, the ratio LF/HF decreased from a baseline value of 3.23 to 2.35 after rTMS. Generally, the ratio LF/HF increased in 60% of all cases.

As can be observed in Table 4, the mean *RR* interval did not change considerably from the baseline phase to the stimulation phase, but the standard deviation increased substantially during rTMS, meaning that dispersion of the points (*RR* values) became apparent. Also, the mean LF component of the spectral curve decreased during the stimulation, so there was a disruption of the normal rhythm of the parasympathetic and sympathetic activities of the cardiovascular system.

The results showed that, for the majority of the cases, a decrease of the *RR* interval was observed, while for 25% of the cases the response was reversed. As an overall, changes in the RR intervals were even more apparent during the first minute of stimulation using trains of 10 Hz.

## 4. Conclusion

With this study we have extended the application field of TMS and ECG integration by examining the effects of rTMS brain stimulation on the heart rhythm as observed through recorded ECG signals. Our findings indicate that it is important to know and understand the basic interactions between the human cortex and the autonomic nervous system. We suggest that ECG monitoring should be performed when stimulating patients through the TMS machine under the repetitive mode of operation, most especially in subjects with known heart ailments or persons in the older age groups. This is essential for checking in real time for any potential changes that could lead to unforeseen events. Our technology will stop stimulation automatically as soon as such initial changes occur. For example, it is reported that subjects older than 40 years of age are more vulnerable to alterations of the cardiac rhythm. If any rTMS session should be undertaken, a monitoring ECG protocol should be followed in order to avoid any complications.

Finally, the monitoring of the HRV is a powerful tool for understanding and monitoring the cardiovascular system, especially for patients with known cardiac illnesses. Objectively, since rTMS has a great impact on some patients suffering from a diverse number of neurological diseases, it remains to be determined if it can also help in predicting any cardiac condition during or after any session of repetitive magnetic stimulation.

## Figures and Tables

**Table 1:** Comparative measurements at baseline and during stimulation.

Subject	Baseline			rTMS stimulation		
number	*RR*	SD1	SD2	*RR*	SD1	SD2
1	932	21.9	62.1	817	21.6	48.3
2	842	46.6	83.9	820	46.7	96.2
3	780	25.6	56.3	767	17	79.8
4	772	16.2	44	615	12.5	74.7
5	937	35.8	85.7	920	36.7	100
6	893	31	77.1	889	33.8	87.1
7	1004	31.7	53.9	982	38.7	79.2
8	872	30.4	63	888	35.9	84.5
9	918	48.1	87.1	959	26.8	59.8
10	668	28.5	100	746	34.6	98

Mean	861.8	31.58	71.31	840.3	30.43	80.76

STD	93.67	9.45	17.03	15.79	10.67	16.64

Total variability	195,846			204,120		

**Table 2:** Influence of rTMS upon the slope of consecutive *RR* intervals during each minute of baseline and rTMS recordings.

Baseline						rTMS				
Min.	1	2	3	4	5	1	2	3	4	5
1	0	0	−1	0	0	0	0	−1	0	1
2	1	0	0	0	0	0	−1	1	1	−1
3	0	0	0	−1	1	0	−1	0	0	0
4	1	0	0	0	0	0	1	0	0	0
5	0	0	0	0	0	1	1	0	1	1
6	0	0	1	0	0	0	0	0	0	1
7	0	0	0	0	0	0	0	0	0	0
8	0	0	0	0	0	0	0	−1	1	0
9	0	0	−1	0	0	−1	1	1	1	−1
10	0	0	1	0	1	1	0	0	0	−1
			∗							∗∗

Baseline: *P < 0.05.

Active phase: **P < 0.02.

**Table 3:** Differences in HRV (as determined from spectral analysis: LF and HF).

Subject	Baseline			rTMS stimulation		
number	LF	HF	L/H	LF	HF	L/H
1	397	159	2.5	167	197	0.69
2	509	1218	**0.41**	742	1153	**0.64**
3	243	167	**1.46**	298	96	**3.12**
4	201	81	**2.48**	143	48	**2.98**
5	824	405	2.04	683	447	1.53
6	967	394	2.45	1470	1400	1.05
7	659	1589	**0.41**	450	538	**0.84**
8	432	271	**1.59**	856	354	**2.42**
9	678	245	2.77	302	261	1.16
10	1619	328	**3.18**	1112	349	**4.94**

Mean	653	486	1.92	622	484	1.94

STD	418	502	1.4	435	447	1.4

**Table 4:** General statistics.

	*RR* (ms)	HR (bpm)	SD1	SD2	LF	HF	L/H
Baseline							
Mean	861	71	32	71	652	489	1.92
±STD	94	4.5	9.5	17	418	502	0.95

rTMS stimulation							
Mean	840	74	30	81	422	484	1.94
±STD	146	12.5	11	17	435	447	1.4

## References

[1] Kobayashi M, Pascual-Leone A (2003). Transcranial magnetic stimulation in neurology. *The Lancet Neurology*.

[2] Hallett M (2000). Transcranial magnetic stimulation and the human brain. *Nature*.

[3] Rossini PM, Rossi S (2007). Transcranial magnetic stimulation: diagnostic, therapeutic, and research potential. *Neurology*.

[4] Siebner HR, Hartwigsen G, Kassuba T, Rothwell JC (2009). How does transcranial magnetic stimulation modify neuronal activity in the brain? Implications for studies of cognition. *Cortex*.

[5] Udupa K, Sathyaprabha TN, Thirthalli J, Kishore KR, Raju TR, Gangadhar BN (2007). Modulation of cardiac autonomic functions in patients with major depression treated with repetitive transcranial magnetic stimulation. *Journal of Affective Disorders*.

[6] Herwig U, Lampe Y, Juengling FD (2003). Add-on rTMS for treatment of depression: a pilot study using stereotaxic coil-navigation according to PET data. *Journal of Psychiatric Research*.

[7] Plewnia C, Pasqualetti P, Grosse S (2014). Treatment of major depression with bilateral theta burst stimulation: a randomized controlled pilot trial. *Journal of Affective Disorders*.

[8] Harel EV, Zangen A, Roth Y, Reti I, Braw Y, Levkovitz Y (2011). H-coil repetitive transcranial magnetic stimulation for the treatment of bipolar depression: an add-on, safety and feasibility study. *The World Journal of Biological Psychiatry*.

[9] Bae EH, Schrader LM, Machii K (2007). Safety and tolerability of repetitive transcranial magnetic stimulation in patients with epilepsy: a review of the literature. *Epilepsy & Behavior*.

[10] Ponnusamy A, Marques JLB, Reuber M (2012). Comparison of heart rate variability parameters during complex partial seizures and psychogenic nonepileptic seizures. *Epilepsia*.

[11] Bauer PR, Kalitzin S, Zijlmans M, Sander JW, Visser GH (2014). Cortical excitability as a potential clinical marker of epilepsy: a review of the clinical application of transcranial magnetic stimulation. *International Journal of Neural Systems*.

[12] Philpott AL, Fitzgerald PB, Cummins TDR, Georgiou-Karistianis N (2013). Transcranial magnetic stimulation as a tool for understanding neurophysiology in Huntington's disease: a review. *Neuroscience and Biobehavioral Reviews*.

[13] Fregni F, Santos CM, Myczkowski ML (2004). Repetitive transcranial magnetic stimulation is as effective as fluoxetine in the treatment of depression in patients with Parkinson's disease. *Journal of Neurology, Neurosurgery and Psychiatry*.

[14] Prikryl R, Ustohal L, Kucerova HP (2014). Repetitive transcranial magnetic stimulation reduces cigarette consumption in schizophrenia patients. *Progress in Neuro-Psychopharmacology and Biological Psychiatry*.

[15] Levkovitz Y, Rabany L, Harel EV, Zangen A (2011). Deep transcranial magnetic stimulation add-on for treatment of negative symptoms and cognitive deficits of schizophrenia: a feasibility study. *International Journal of Neuropsychopharmacology*.

[16] Frantseva M, Cui J, Farzan F, Chinta LV, Velazquez JLP, Daskalakis ZJ (2014). Disrupted cortical conductivity in schizophrenia: TMS-EEG study. *Cerebral Cortex*.

[17] Julkunen P, Jauhiainen AM, Westerén-Punnonen S (2008). Navigated TMS combined with EEG in mild cognitive impairment and Alzheimer's disease: a pilot study. *Journal of Neuroscience Methods*.

[18] Rossini PM, Rossi S, Babiloni C, Polich J (2007). Clinical neurophysiology of aging brain: from normal aging to neurodegeneration. *Progress in Neurobiology*.

[19] Hummel FC, Cohen LG (2006). Non-invasive brain stimulation: a new strategy to improve neurorehabilitation after stroke?. *The Lancet Neurology*.

[20] Yozbatiran N, Alonso-Alonso M, See J (2009). Safety and behavioral effects of high-frequency repetitive transcranial magnetic stimulation in stroke. *Stroke*.

[21] Fregni F, Boggio PS, Valle AC (2006). A sham-controlled trial of a 5-day course of repetitive transcranial magnetic stimulation of the unaffected hemisphere in stroke patients. *Stroke*.

[22] Sokhadze EM, El-Baz A, Baruth J, Mathai G, Sears L, Casanova MF (2009). Effects of low frequency repetitive transcranial magnetic stimulation (rTMS) on gamma frequency oscillations and event-related potentials during processing of illusory figures in Autism. *Journal of Autism and Developmental Disorders*.

[23] Bloch Y, Harel EV, Aviram S, Govezensky J, Ratzoni G, Levkovitz Y (2010). Positive effects of repetitive transcranial magnetic stimulation on attention in ADHD Subjects: a randomized controlled pilot study. *The World Journal of Biological Psychiatry*.

[24] Acosta MT, Leon-Sarmiento FE (2003). Repetitive transcranial magnetic stimulation (rTMS): new tool, new therapy and new hope for ADHD. *Current Medical Research and Opinion*.

[25] Konen CS, Haggard P (2014). Multisensory parietal cortex contributes to visual enhancement of touch in humans: a single-pulse TMS study. *Cerebral Cortex*.

[26] Paus T, Castro-Alamancos MA, Petrides M (2001). Cortico-cortical connectivity of the human mid-dorsolateral frontal cortex and its modulation by repetitive transcranial magnetic stimulation. *European Journal of Neuroscience*.

[27] Berardelli A, Inghilleri M, Rothwell JC (1998). Facilitation of muscle evoked responses after repetitive cortical stimulation in man. *Experimental Brain Research*.

[28] Wassermann EM (1998). Risk and safety of repetitive transcranial magnetic stimulation: report and suggested guidelines from the International Workshop on the Safety of Repetitive Transcranial Magnetic Stimulation, June 5–7, 1996. *Electroencephalography and Clinical Neurophysiology/Evoked Potentials*.

[29] Rossi S, Hallett M, Rossini PM, Pascual-Leone A (2009). Safety, ethical considerations, and application guidelines for the use of transcranial magnetic stimulation in clinical practice and research. *Clinical Neurophysiology*.

[30] Bastani A, Jaberzadeh S (2012). Does anodal transcranial direct current stimulation enhance excitability of the motor cortex and motor function in healthy individuals and subjects with stroke: a systematic review and meta-analysis. *Clinical Neurophysiology*.

[31] Manganotti P, Formaggio E, Storti SF (2013). Effect of high-frequency repetitive transcranial magnetic stimulation on brain excitability in severely brain-injured patients in minimally conscious or vegetative state. *Brain Stimulation*.

[32] Barron W, Coote JH (1973). The contribution of articular receptors to cardiovascular reflexes elicited by passive limb movement. *Journal of Physiology*.

[33] Zipes DP (2008). Heart-brain interactions in cardiac arrhythmias: role of the autonomic nervous system. *Cleveland Clinic Journal of Medicine*.

[34] Engström G, Hedblad B, Juul-Möller S, Tydén P, Janzon L (2000). Cardiac arrhythmias and stroke: increased risk in men with high frequency of atrial ectopic beats. *Stroke*.

[35] Akashi YJ, Nef HM, Möllmann H, Ueyama T (2010). Stress cardiomyopathy. *Annual Review of Medicine*.

[36] Yoshida T, Yoshino A, Kobayashi Y, Inoue M, Kamakura K, Nomura S (2001). Effects of slow repetitive transcranial magnetic stimulation on heart rate variability according to power spectrum analysis. *Journal of the Neurological Sciences*.

[37] Clarke BM, Upton ARM, Kamath MV, Al-Harbi T, Castellanos CM (2006). Transcranial magnetic stimulation for migraine: clinical effects. *Journal of Headache and Pain*.

[38] Peters JC, Reithler J, Schuhmann T (2013). On the feasibility of concurrent human TMS-EEG-fMRI measurements. *Journal of Neurophysiology*.

[39] Pellicciari MC, Brignani D, Miniussi C (2013). Excitability modulation of the motor system induced by transcranial direct current stimulation: a multimodal approach. *NeuroImage*.

[40] Pascual-Leone A, Freitas C, Oberman L (2011). Characterizing brain cortical plasticity and network dynamics across the age-span in health and disease with TMS-EEG and TMS-fMRI. *Brain Topography*.

[41] Adjouadi M, Cabrerizo M, Rojas N, Perez JO Electrocardiography Triggered Transcranial Magnetic Stimulation Systems and Methods.

[42] Gulli G, Tarperi C, Cevese A, Acler M, Bongiovanni G, Manganotti P (2013). Effects of prefrontal repetitive transcranial magnetic stimulation on the autonomic regulation of cardiovascular function. *Experimental Brain Research*.

[43] Luo Y, Hargraves RH, Belle A (2013). A hierarchical method for removal of baseline drift from biomedical signals: application in ECG analysis. *The Scientific World Journal*.

[44] Brennan M, Palaniswami M, Kamen P (2001). Do existing measures of Poincaré plot geometry reflect nonlinear features of heart rate variability?. *IEEE Transactions on Biomedical Engineering*.

[45] Chess GF, Tam RM, Calaresu FR (1975). Influence of cardiac neural inputs on rhythmic variations of heart period in the cat. *The American Journal of Physiology*.

[46] Kamath MV, Fallen EL (1993). Power spectral analysis of heart rate variability—a noninvasive signature of cardiac autonomic function. *Critical Reviews in Biomedical Engineering*.

[47] Guyton A, Hall J (1991). Nervous regulation of the circulation, and rapid control of arterial pressure. *Textbook of Medical Physiology*.

[48] Camm AJ, Malik M, Bigger JT (1996). Heart rate variability—standards of measurement, physiological interpretation, and clinical use. *Circulation*.

